# Supplementary material for: Validation of an Analytical Method of 3′,4′,5-Trihydroxy-3-Methoxy-6,7-Methylenedioxyflavone 4′-Glucuronide for Standardization of Spinacia oleracea
Source: Molecules. 2024 May 24;29(11):2494. doi: 10.3390/molecules29112494 (PMC11173439; doi:10.3390/molecules29112494)
Supplement: Supplementary file 1 [file molecules-29-02494-s001.zip › molecules-2985764-supplementary.pdf]

Supplementary materials

Validation of an Analytical Method of 3',4',5-Trihydroxy-3-Methoxy-6,7-Methylenedioxyflavone 4'-Glucuronide for Standardization of *Spinacia oleracea*

Yun Gon Son[1], Juyoung Jung[2], Dong Kun Lee[3], Sang Won Park[4], Jeong Yoon Kim[1,*], Hyun Joon Kim[5,*]

List of contents

- **Table S1**. Instrumental parameters of HPLC and Q-TOF/MS
- **Figure S1 and S2**. HPLC chromatogram of spinach samples by different wavelength
- **Figure S3 and S4**. HPLC chromatogram of spinach samples by different analytical methods
- **Figure S5**. HPLC chromatogram of spinach samples by different column conditions
- **Figure S6**. HPLC chromatogram of spinach sample and authentic samples
- **Figures S7, S8, Tables S2, and S3**. Quantitative analysis results of six metabolites (**1**-**6**) in spinach samples based on the molar extinction coefficient of TMG
- **Figure S9**. Residuals plot of TMG
- **Table S4–S8** ANOVA test of each samples

Table S1. Instrumental parameters of HPLC and Q-TOF/MS

HPLC	
Instrument	Agilent 1260 HPLC
Flow rate	1.0 ml min^{-1}
Injection volume	10 µl
Solvent A	Water containing 0.1% acetic acid
Solvent B	ACN containing 0.1% acetic acid
Column	X-Bridge C18, 4.6 x 150 mm, 5 um (waters)
Q-TOF/MS	
LC instrument	NEXERA UHPLC, Shimadzu
Q-TOF/MS instrument	X500R TOF/MS, SCIEX
Flow rate	0.3 ml/min
Injection volume	1 µl
Solvent A	Water containing 0.1% acetic acid
Solvent B	ACN containing 0.1% acetic acid
Ion source	
- Source name	TurboIonSpray
- Curtain gas (psi)	30
- Ion source gas 1 (psi)	50
- Ion source gas 2 (psi)	50
- Temperature (ºC)	450
Experiment	
- Polarity	Positive
- Ionspray voltage (V)	5500
- TOF start mass (Da)	100
- TOF stop mass (Da)	10000
- Accumulation time (s)	0.1
- Declustering potential (V)	50
- Collision energy (V)	35

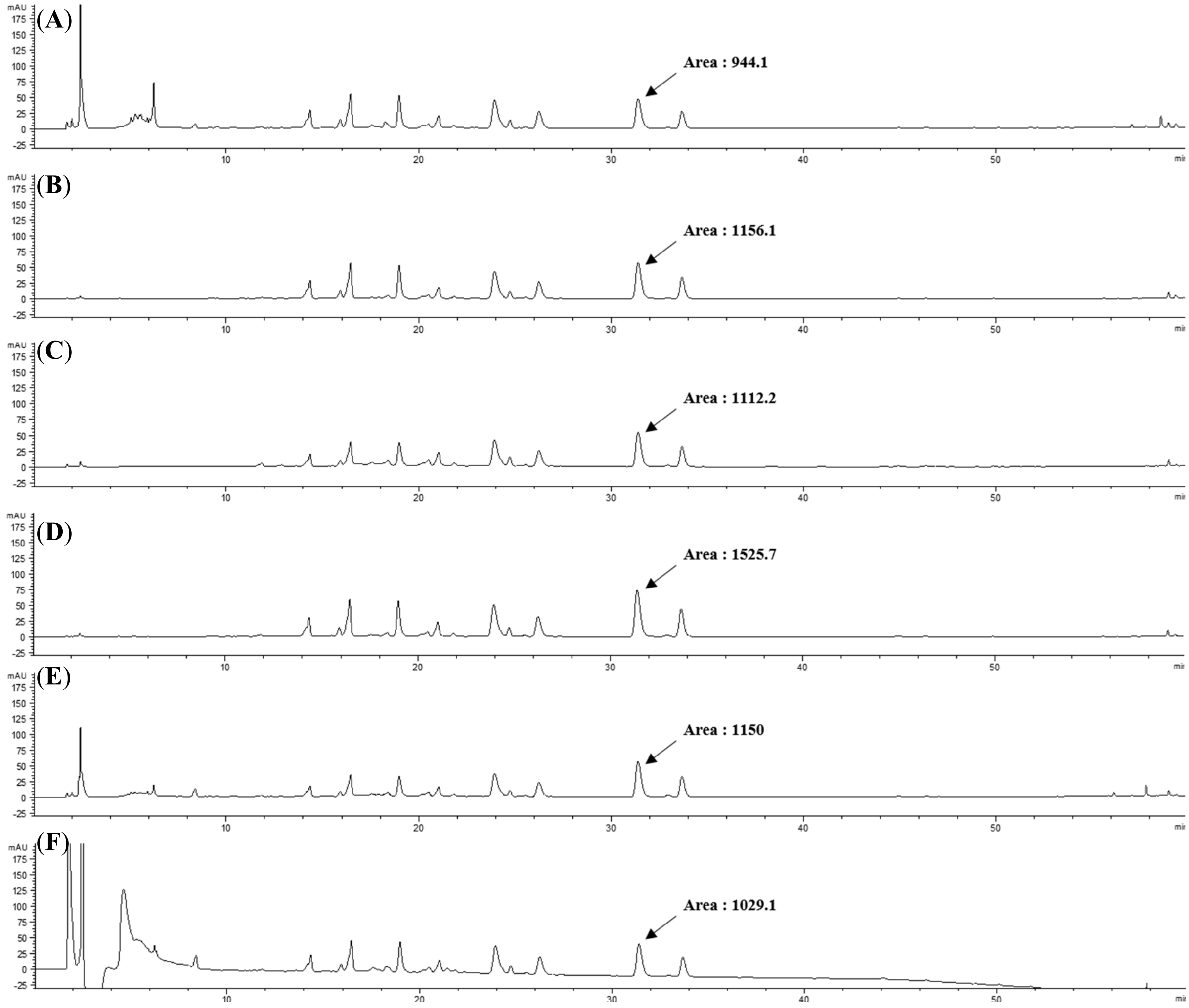

Figure S1. HPLC chromatogram of freeze-dried spinach (A) 254 nm, (B) 360 nm, (C) 320 nm, (D) 350 nm, (E) 280 nm, (F) 230 nm

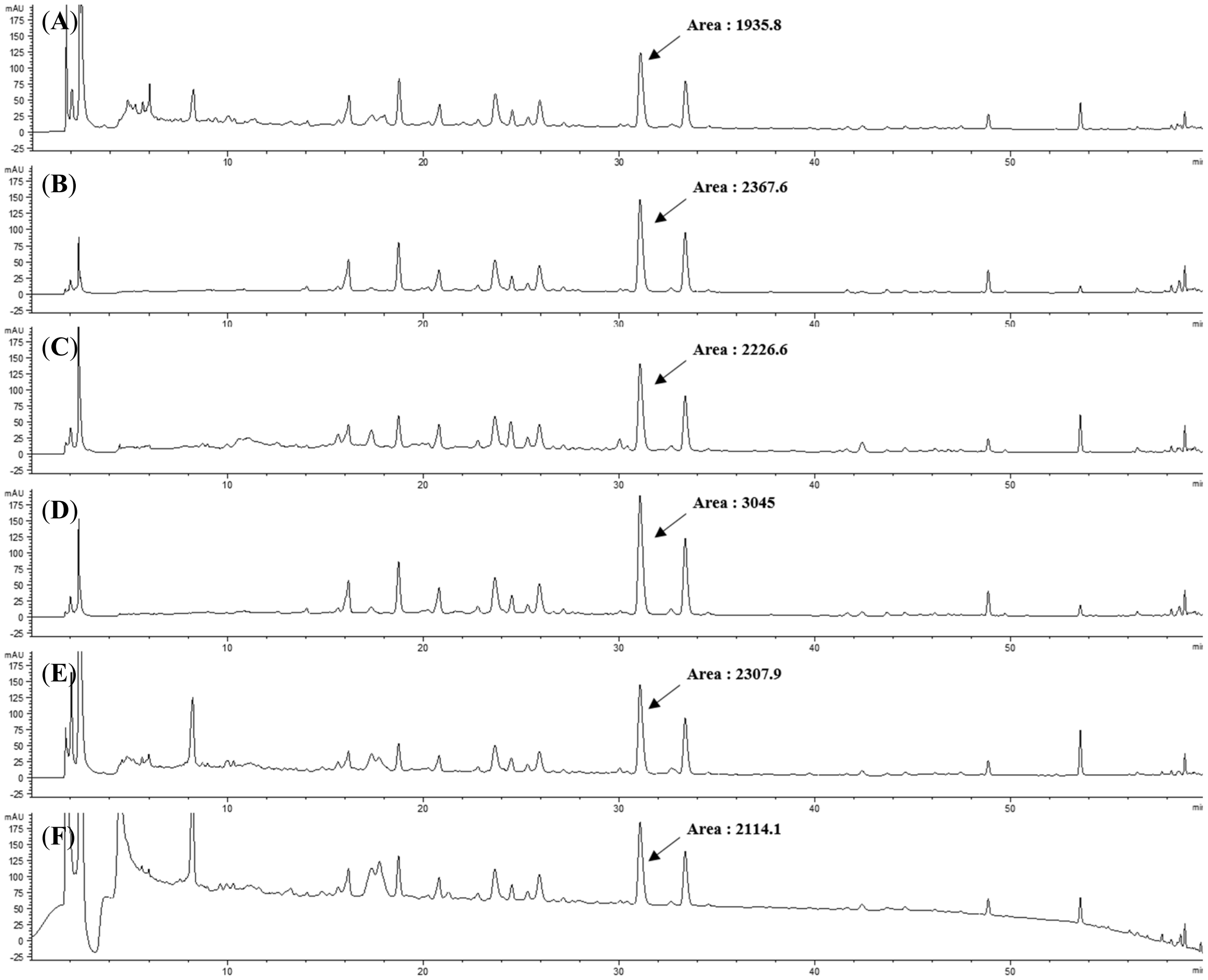

Figure S2. HPLC chromatogram of spinach extract concentrate (A) 254 nm, (B) 360 nm, (C) 320 nm, (D) 350 nm, (E) 280 nm, (F) 230 nm

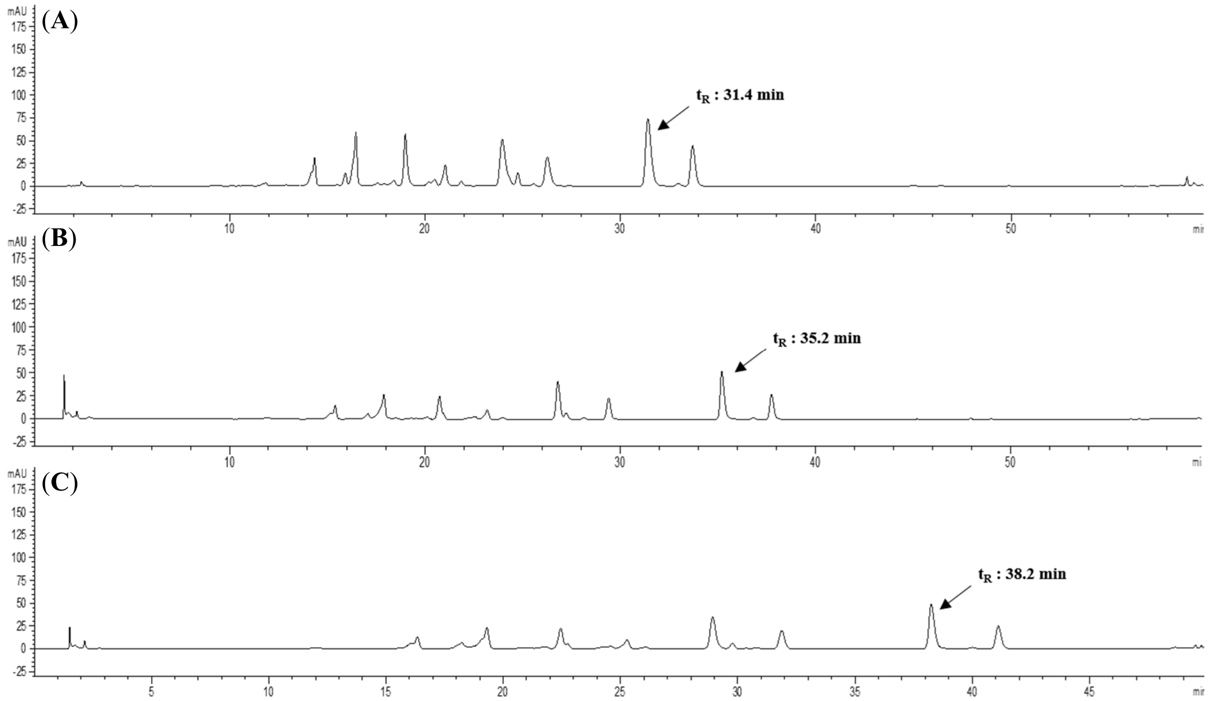

Figure S3. HPLC chromatogram (350 nm) of different methods analysis freeze-dried spinach (A) experimental methods and change the method of (B) the ratio of solvent B, (C) running time

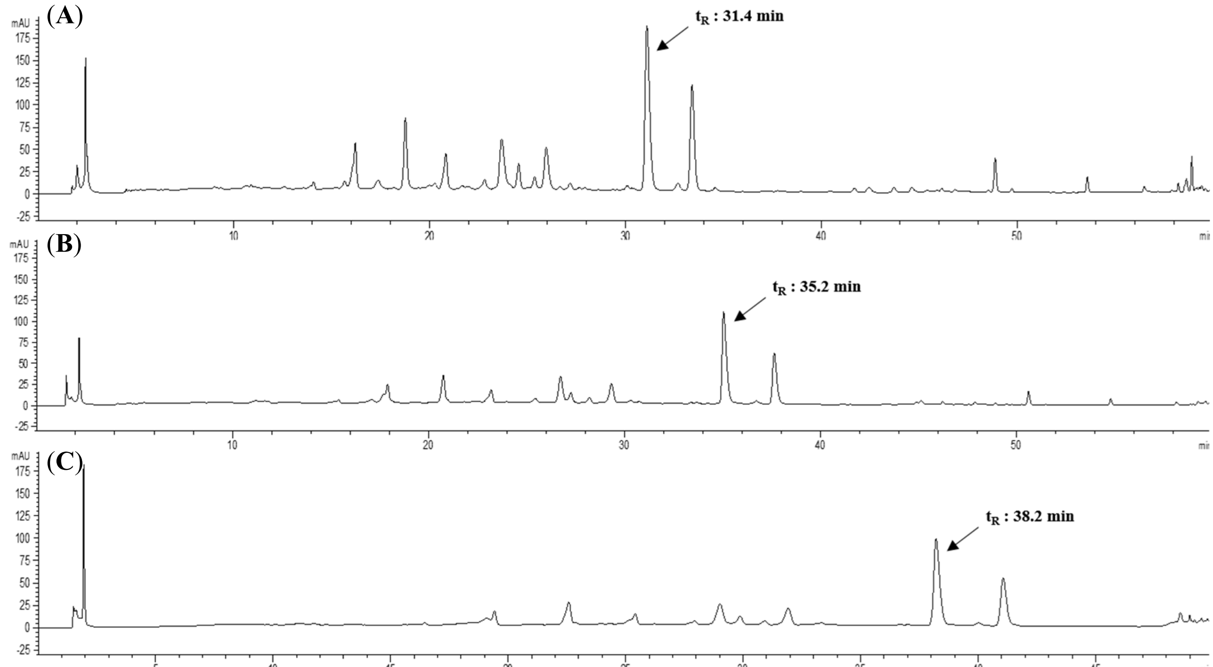

Figure S4. HPLC chromatogram (350 nm) of different methods analysis spinach extract concentrate (A) experimental methods and change the method of (B) the ratio of solvent B, (C) running time

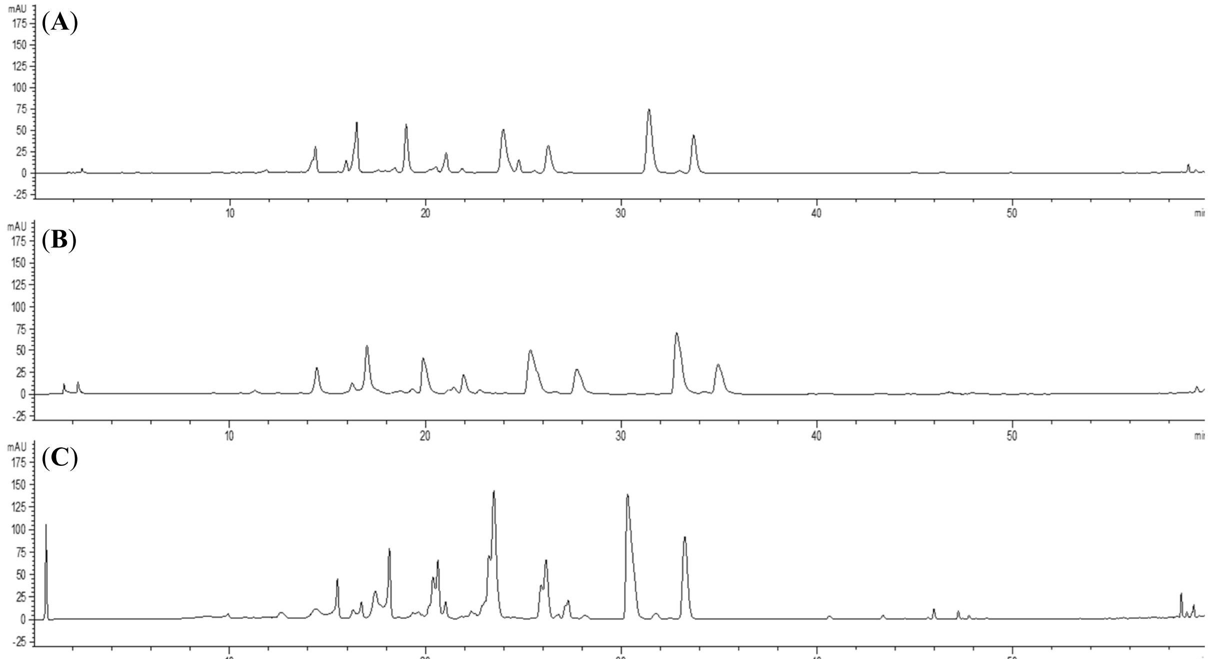

Figure S5. HPLC chromatogram (350 nm) of different column analysis freeze-dried spinach (A) Waters X-Bridge C18 (4.6 x 150 mm, 5 μm) column (B) Waters X-Bridge C18 (4.6 x 250 mm, 5 μm) column, (C) Agilent Poroshell 120 EC C18 (2.1 x 100 mm, 2.7 μm) column

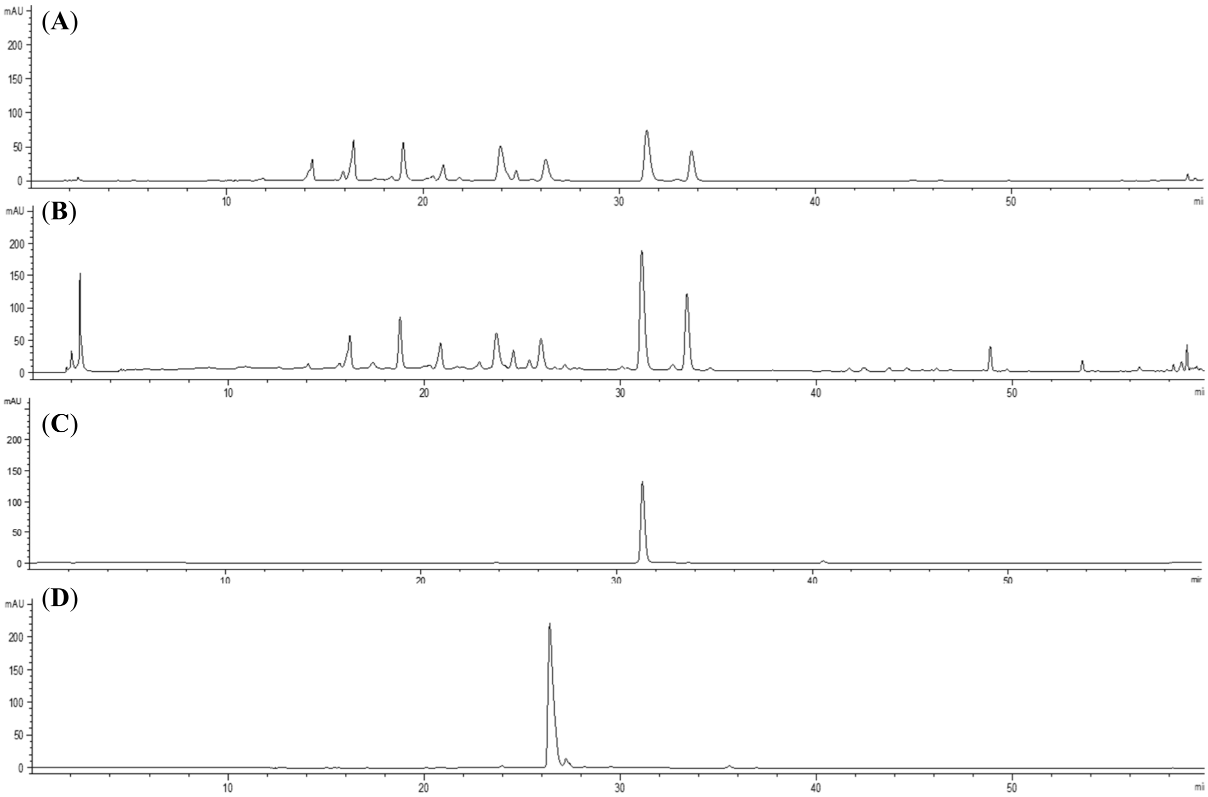

Figure S6. HPLC chromatogram (350 nm) of (A) freeze-dried spinach, (B) spinach extract concentrate, (C) the standard (TMG) solution at 62.5 µg/ml, (D) spinatoside solution at 200 µg/ml

Table S2. Flavonoids content of FDS (**A**) spinacetin 3-O-glucosyl-(1→6)-[apiosyl(1→2)]-glucoside; (**B**) spinacetin-3-O-glucosyl-(1→6)-glucoside (**C**) spinatoside; (**D**) jaceidin 4'-glucuronide; (E) 3',4',5-trihydroxy-3-methoxy-6,7-methylenedioxyflavone 4'-glucuronide; (**F**) 5,4'-dihydroxy-3,3'-dimethoxy-6,7-methylendixoyflavone-4'-glucuronide based on the molar extinction coefficient of TMG.

Compound	Area	ppm	content
A	766.4	23.3	465.9
B	699.6	21.3	426.2
C	1086.7	32.8	656.7
D	590.7	18.1	361.3
E	1525.7	45.9	918.1
F	775.1	23.6	471.1

Table S3. Flavonoids content of SEC (**A**) spinacetin 3-O-glucosyl-(1→6)-[apiosyl(1→2)]-glucoside; (**B**) spinacetin-3-O-glucosyl-(1→6)-glucoside (**C**) spinatoside; (**D**) jaceidin 4'-glucuronide; (E) 3',4',5-trihydroxy-3-methoxy-6,7-methylenedioxyflavone 4'-glucuronide; (**F**) 5,4'-dihydroxy-3,3'-dimethoxy-6,7-methylendixoyflavone-4'-glucuronide based on the molar extinction coefficient of TMG.

Compound	Area	ppm	content
A	688.7	21.0	419.7
B	891.9	27.0	540.7
C	1039.3	31.4	628.5
D	704.6	21.5	429.1
E	3045.0	91.1	1823.0
F	1609.9	48.4	968.3

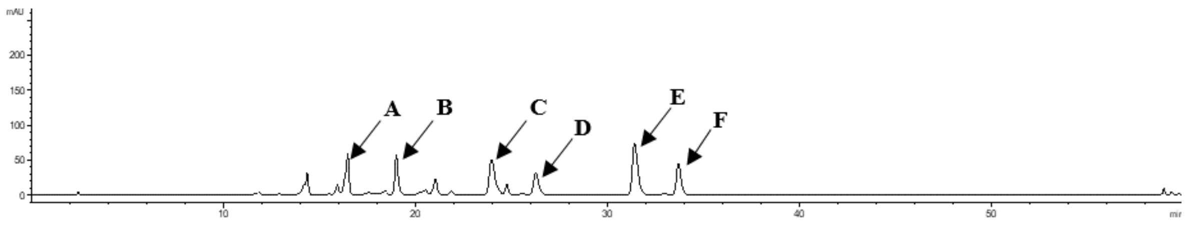

Figure S7. HPLC chromatogram of FDS (**A**) spinacetin 3-O-glucosyl-(1→6)-[apiosyl(1→2)]-glucoside; (**B**) spinacetin-3-O-glucosyl-(1→6)-glucoside (**C**) spinatoside; (**D**) jaceidin 4'-glucuronide; (E) 3',4',5-trihydroxy-3-methoxy-6,7-methylenedioxyflavone 4'-glucuronide; (**F**) 5,4'-dihydroxy-3,3'-dimethoxy-6,7-methylendixoyflavone-4'-glucuronide

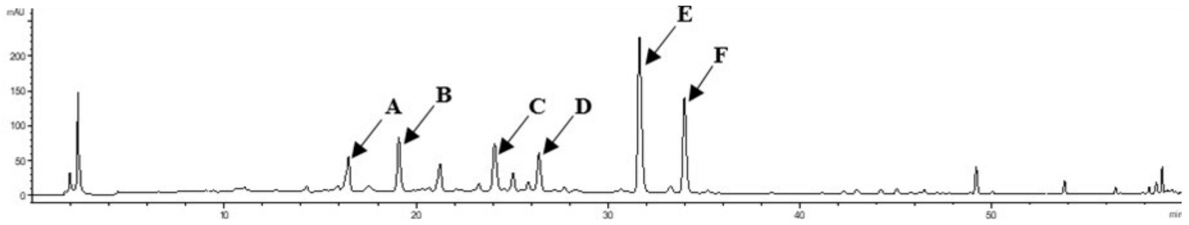

Figure S8. HPLC chromatogram of SEC (**A**) spinacetin 3-O-glucosyl-(1→6)-[apiosyl(1→2)]-glucoside; (**B**) spinacetin-3-O-glucosyl-(1→6)-glucoside (**C**) spinatoside; (**D**) jaceidin 4'-glucuronide; (E) 3',4',5-trihydroxy-3-methoxy-6,7-methylenedioxyflavone 4'-glucuronide; (**F**) 5,4'-dihydroxy-3,3'-dimethoxy-6,7-methylendixoyflavone-4'-glucuronide

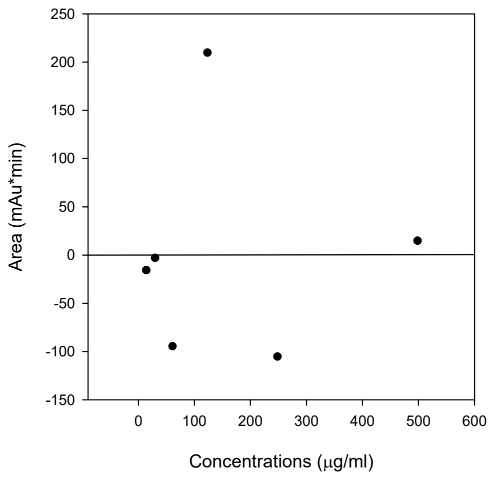

Figure S9. Residuals plot of TMG

Table S4. ANOVA test of intra-day precision for FDS at 0.5 g

	n	Average	SD	F	p
Day 1[a]	5	1105	7.5	2.076	0.122
Day 2[b]	5	1112	8.0		
Day 3[c]	5	1109	2.6		
Day 4[d]	5	1119	1.9		
Day 5[e]	5	1106	3.4		

Table S5. ANOVA test of intra-day precision for FDS at 1.5 g

	n	Average	SD	F	p
Day 1[a]	5	1089	1.2	1.67	0.197
Day 2[b]	5	1089	6.2		
Day 3[c]	5	1094	0.6		
Day 4[d]	5	1097	0.3		
Day 5[e]	5	1089	1.0		

Table S6. ANOVA test of intra-day precision for SEC at 0.5 g

	n	Average	SD	F	p
Day 1[a]	5	2606	44	0.16	0.999
Day 2[b]	5	2604	43		
Day 3[c]	5	2600	42		
Day 4[d]	5	2602	44		
Day 5[e]	5	2605	36		

Table S7. ANOVA test of intra-day precision for SEC at 1.5 g

	n	Average	SD	F	p
Day 1[a]	5	2570	64	0.376	0.823
Day 2[b]	5	2582	51		
Day 3[c]	5	2582	55		
Day 4[d]	5	2608	43		
Day 5[e]	5	2594	51		

Table S8. ANOVA test of linearity for TMG concentration

Concentrate(μg/ml)	n	Average	SD	F	p
15.625	3	487.1	6.7	22359.2	0
31.25	3	1018.4	10.0		
62.5	3	1956.7	28.1		
125	3	4428.2	68.8		
250	3	8269.4	119.4		
500	3	16911.0	108.4		
